# IRE1α Disruption Causes Histological Abnormality of Exocrine Tissues, Increase of Blood Glucose Level, and Decrease of Serum Immunoglobulin Level

**DOI:** 10.1371/journal.pone.0013052

**Published:** 2010-09-27

**Authors:** Takao Iwawaki, Ryoko Akai, Kenji Kohno

**Affiliations:** 1 Iwawaki Initiative Research Unit, RIKEN, Wako, Saitama, Japan; 2 PRESTO, Japan Science and Technology Agency, Kawaguchi, Saitama, Japan; 3 Laboratory of Molecular and Cell Genetics, Nara Institute of Science and Technology, Ikoma, Nara, Japan; Texas A&M University, United States of America

## Abstract

Accumulation of unfolded proteins in the endoplasmic reticulum (ER) causes ER stress. As a cellular adaptive response to ER stress, unfolded protein response (UPR) activates molecules for the quality control of ER proteins. One enzyme that plays an important role in UPR is Inositol Requiring Enzyme-1 (IRE1), which is highly conserved from yeast to humans. In particular, mammalian IRE1α activates X-box-binding protein 1 (XBP1) by unconventional splicing of XBP1 mRNA during ER stress. From analysis of knockout mice, both IRE1α and XBP1 have been shown to be essential for development and that XBP1 is necessary for the secretory machinery of exocrine glands, plasma cell differentiation, and hepatic lipogenesis. However, the essentiality of IRE1α in specific organs and tissues remains incompletely understood. Here, we analyzed the phenotype of *IRE1α* conditional knockout mice and found that *IRE1α*-deficient mice exhibit mild hypoinsulinemia, hyperglycemia, and a low-weight trend. Moreover, IRE1α disruption causes histological abnormality of the pancreatic acinar and salivary serous tissues and decrease of serum level of immunoglobulin produced in the plasma cells, but not dysfunction of liver. Comparison of this report with previous reports regarding *XBP1* conditional knockout mice might provide some clues for the discovery of the novel functions of IRE1α and XBP1. (196 words)

## Introduction

Since the majority of secretory proteins, such as antibodies, digestive enzymes, and hormones, are synthesized in the cytoplasm and are cotranslationally translocated into the lumen of the endoplasmic reticulum (ER) through a narrow channel called translocon on the ER membrane, they are initially located in the ER as unfolded and unmodified nascent polypeptides. These proteins then undergo meticulous folding by molecular chaperones, correct disulfide bond formation by protein disulfide isomerases, and proper oligosaccharide modification by the oligosaccharyltransferase complex, sugar trimming enzymes, and calnexin/calreticulin cycle in the ER [1], [2]. Therefore, when cells produce these proteins in large amounts, the ER is thought to be liable to become overloaded for the maturation of these proteins. Accumulation of unfolded proteins in the ER also causes ER stress. To adaptively respond to ER stress, the cell induces the transcriptional activation of molecules for the maturation of proteins in the ER. This response is called unfolded protein response (UPR) [3]. Thus, UPR is an important cellular response for the mass production of functional secretory proteins from unfolded proteins in cells which produce them in large amounts.

To date, several molecules have been reported to play important roles in UPR. One of these molecules, IRE1, is an ER-located type I transmembrane protein with a kinase domain and RNase domain in the cytosolic region. When exposed to ER stress, via *trans*-autophosphorylation and activation of its RNase domain, IRE1 induces unconventional splicing of an mRNA encoding a specific transcription factor responsible for UPR activation [4]–[8]. IRE1 is highly conserved from yeast to humans, and two IRE1 paralogues have been reported in mammals: IRE1α and IRE1β [9]–[11]. Specifically, IRE1α induces the unconventional splicing of XBP1 mRNA under ER stress condition [12]. The spliced XBP1 mRNA is then translated into a functional transcription factor to induce UPR. Besides IRE1, two ER-located transmembrane proteins, PERK and ATF6, play important roles in mammalian UPR [13], [14]. On sensing ER stress, PERK induces the phosphorylation of eIF2α and the translational activation of ATF4 [15]. On the other hand, under ER stress condition, ATF6 is cleaved by Site-1 and Site-2 proteases, and its cytoplasmic domain is translocated to the nucleus [16], [17]. Both ATF4 and the cleaved ATF6 work as transcription factors in UPR induction, as well as XBP1 which is activated by IRE1α.

As described above, IRE1α directly catalyzes the cleavage of XBP1 mRNA in the splicing reaction under ER stress condition [12]. To our knowledge, this reaction is exclusively dependent on IRE1α activity and is not detected in *IRE1α*-deficient cells [18]. This implies that IRE1α and XBP1 function on the same signal transduction pathway in ER stress response. Also, *IRE1α* knockout (KO) mice and *XBP1* KO mice commonly have embryonic lethality and that both IRE1α and XBP1 play an essential role in mammalian development [19]–[21]. However, although embryonic lethality of *XBP1* KO mice is rescued with an *XBP1* transgene specifically expressed in the liver [22], that of *IRE1α* KO mice is rescued with endogenous IRE1α specifically expressed in the extra-embryonic tissues and not in the liver [18]. This suggests that not only a known IRE1α-dependent XBP1 function but also an XBP1-independent IRE1α function(s) may exists in extra-embryonic tissues and that an IRE1α-independent XBP1 function(s) may exists in the fetal liver. Thus, a comparison analysis of conventional and conditional KO mice in terms of IRE1α and XBP1 may further provide some clues for the discovery of additional tissue-specific functions of each molecule. Analysis of *XBP1* conditional KO mice, including *XBP1* KO mice rescued with an *XBP1* transgene specifically expressed in the liver, previously demonstrated that XBP1 is required for the secretory machinery of exocrine glands, plasma cell differentiation, and hepatic lipogenesis [22]–[24]. However, it remains unclear whether IRE1α plays an essential function for these biological phenomena. To elucidate this, we analyzed the phenotype of *IRE1α* conditional KO mice in this study.

## Methods

### IRE1α conditional KO mice

As previously described, we generated viable *IRE1α* conditional KO mice (*Mox2^+/Cre^*; *IRE1α^ΔNeo/ΔR^*) and control mice (*Mox2^+/+^*; *IRE1α^ΔNeo/ΔR^*) by breeding *Mox2^+/Cre^*; *IRE1α^+/ΔR^* mice with *Mox2^+/+^*; *IRE1α^ΔNeo/ΔNeo^* mice [18]. *IRE1α* conditional KO mice and control mice were born at near-Mendelian ratios. All mice used in the experiment were maintained on a mixed (C57BL/6 x 129/SvE) background. Experimental protocols involving animals were approved by Animal Studies Committees at RIKEN (the permit number; H22-1-105) and NAIST (the permit number; 1011).

### Measurement of blood glucose and insulin

Blood glucose level was measured using a portable glucose measuring device (Arkray). Insulin level was determined by enzyme linked immunosorbent assay (ELISA) using mouse insulin as a standard (Shibayagi). Glucose tolerance tests were performed on 20-week-old *IRE1α* conditional KO and control mice that had been fasted for 16 hours. Mice were orally administered with 2 mg/g body weight glucose. Blood glucose level and serum insulin level were measured at indicated intervals.

### Histological analysis

Each tissue was fixed in 10% formalin and then embedded in paraffin. Paraffin blocks were sliced into 5-µm-thick sections and stained with hematoxylin and eosin for general histopathological analysis. Immunohistochemical analysis was performed using 6-µm-thick paraffin sections. Immunoreactivity of insulin and glucagons was detected using guinea pig polyclonal anti-insulin and rabbit polyclonal anti-glucagon antibodies (both from Dako), respectively. Immunoreaction with secondary antibodies and chromogenic reaction with 3,3′-diaminobenzidine tetrahydrochloride were performed according to standard procedures.

### Western blot analysis

The tissues were lysed in SDS sample buffer (50 mM Tris-HCl pH 6.8, 2% SDS, 50 mM DTT, 10% glycerol, and 1 µg/ml bromophenol blue), and the lysate was heated to 98°C for 10 min. SDS-PAGE (10% gel) was used to resolve the proteins in the lysate. After electrophoresis, the proteins were electrotransferred onto polyvinylidene fluoride microporous membranes. Immunodetection of amylase and glyceraldehyde 3-phosphate dehydrogenase (GAPDH) was performed using rabbit polyclonal anti-amylase antibody (Sigma) and mouse monoclonal anti-GAPDH antibody (Cell signaling), respectively.

### Analysis of B cell differentiation

Serum levels of IgM and IgG1 were measured by ELISA (Bethyl Laboratories Inc.) according to the manufacturer's instruction. Flow cytometry analysis was performed with FACS vantage SE (Becton Dickinson) and FlowJo ver. 6.3.2 software. For the analysis, single-cell suspensions from the spleen were prepared according to the protocol of Beckman Coulter Japan. Splenocytes were then stained with PE-conjugated IgD antibody (Becton Dickinson), FITC-conjugated IgM antibody (eBioscience), and APC-conjugated B220 antibody (Becton Dickinson).

### Measurement of transaminase activity in serum

Both aspartate aminotransferase (AST) and alanine aminotransferase (ALT) activities in serum were measured using the transaminase assay kit (Wako).

### Quantitative PCR analysis

Quantitative PCR analysis of each transcript was performed using TaqMan probe and 7900HT (Applied Biosystems) in accordance with the manufacturer's instructions with GAPDH transcript as an internal control. Results are expressed as mean ± S.E.M from triplicate experiments using RNA isolated from three independent tissues. Each probe/primer set, namely, Mm00469005_m1, Mm00499536_m1, Mm00772290_m1, and Mm01204691_m1 (all from Applied Biosystems) was used for the quantification of IRE1β, diacylglycerol O-acyltranserase 2 (Dgat2), stearoyl-coenzyme A desaturase 1 (Scd1), and acetyl-coenzyme A carboxylase 2 (Acc2) transcripts, respectively. IRE1α transcripts were quantified using the forward primer: 5′-ggt cca atc gta cgg cag tt-3′, the reverse primer: 5′-tct ctc aca gag cca cct ttg tag-3′, and probe: 5′-FAM-cct gca gac aga tct-MGB-3′. Total XBP1 transcripts were quantified using the forward primer: 5′-gaa tgg aca cgc tgg atc ct-3′, the reverse primer: 5′-gcc acc agc ctt act cca ctc-3′, and probe: 5′-FAM-cct ctg gaa cct cg-MGB-3′. Spliced XBP1 transcripts were quantified using the forward primer: 5′-gaa tgg aca cgc tgg atc ct-3′, the reverse primer: 5′-cag agt cca tgg gaa gat gtt ct-3′, and probe: 5′-FAM-cac ctg ctg cgg act-MGB-3′.

## Results

### IRE1α-deficient mice exhibit mild hypoinsulinemia, hyperglycemia, and low-weight trend

**Figure 1:**
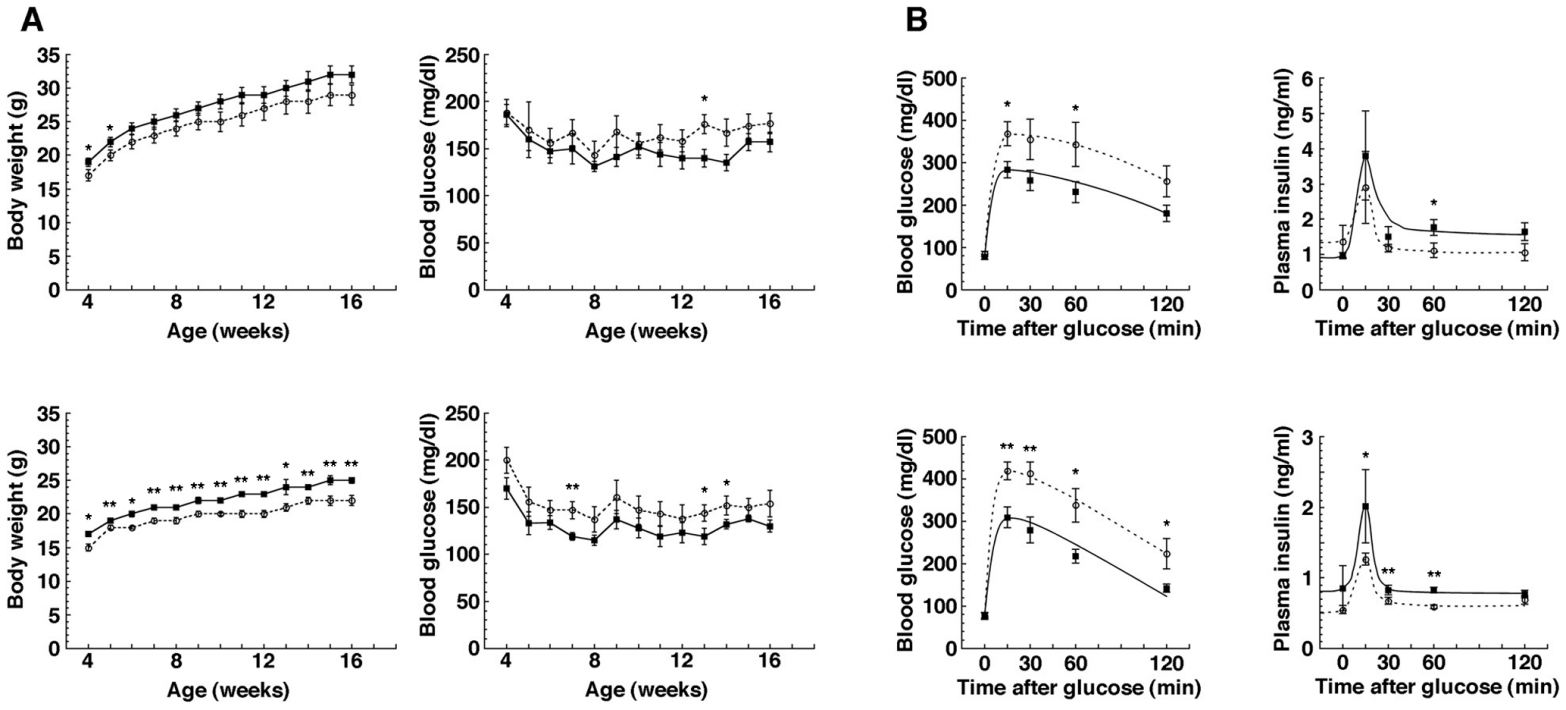
Comparison of body weight, blood glucose level, and plasma insulin level. (**A**) Weekly changes in body weight (left) and blood glucose level (right) under an ad libitum feeding condition between 4 and 16 weeks after birth. The solid line with squares and the broken line with circles refer to the control and *IRE1α* CKO mice data, respectively. The upper figures and lower figures refer to the data for male and female mice, respectively. The plots indicate mean and error bars denote S.E.M (n = 7–10). Statistical significance of differences between control and *IRE1α* CKO mice was determined by Student's t-test (*p<0.05, **p<0.01). (**B**) Changes in blood glucose level (left) and blood insulin level (right) after oral administration of glucose. The solid line with squares and the broken line with circles refer to the control and *IRE1α* CKO mice data, respectively. The upper figures and lower figures refer to the data for male and female mice, respectively. Measurement was performed at 20 weeks old. The plots indicate mean and error bars denote S.E.M (n = 7–10). Statistical significance of differences between control and *IRE1α* CKO mice was determined by Student's t-test (*p<0.05, **p<0.01).

**Figure 2:**
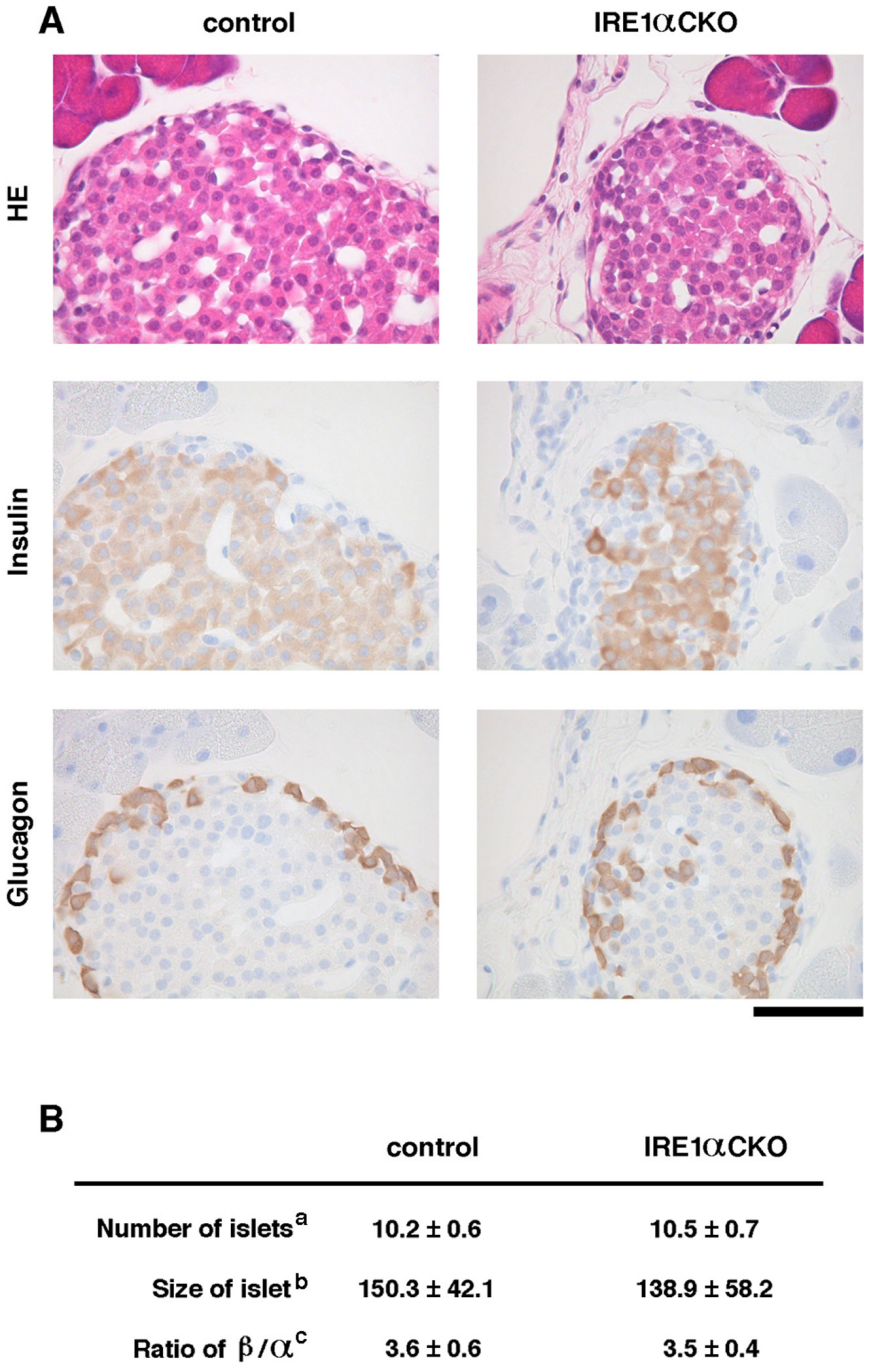
Histological analysis of the islets in *IRE1α* CKO mice and control mice. (**A**) The upper, middle, and lower panels show HE-stained sections, immunostained images using anti-insulin antibodies, and immunostained images using anti-glucagon antibodies, respectively. The three images for the control or *IRE1α* CKO mice are derived from the serial sections (scale bar: 50 µm). (**B**) ^a^The number of islets counted per 1 cm^2^ of pancreas section is shown as mean ± S.E.M (n = 10 pancreas sections). ^b^The diameter (μm) of islet is shown as mean ± S.E.M (n = 50). ^c^The ratio of insulin^+^ cells to glucagons^+^ cells per islet is shown as mean ± S.E.M (n = 10). All the data were obtained from 20 weeks old female mice.

Although *IRE1α* conventional KO mice show embryonic lethality as described earlier, embryo proper-restricted *IRE1α* conditional KO mice, which specifically express IRE1α in the extra-embryonic tissues, can avoid embryonic lethality [18], [20], [21]. We initially measured body weight and blood glucose level from age 4 weeks to age 16 weeks to examine the growth process and health condition of embryo proper-restricted *IRE1α* conditional KO mice (hereinafter *IRE1α* CKO mice). *IRE1α* CKO mice exhibited mild hyperglycemia and a low-weight trend compared with control mice under free feeding conditions (Figure 1A), although there was no difference in body length, amount of feed and water intake per day between *IRE1α* CKO and control mice (Table S1). To investigate the blood glucose regulatory ability of *IRE1α* CKO mice, we performed oral glucose tolerance test (OGTT). The measurement of blood insulin level during OGTT revealed that insulin level at its peak was decreased by 30∼50% in *IRE1α* CKO mice compared with control mice. On the other hand, the measurement of blood glucose level during OGTT showed that *IRE1α* CKO mice have a significantly higher blood glucose level at its peak than control mice and that *IRE1α* CKO mice required a longer time for blood glucose level to recover than control mice (Figure 1B). Moreover, we measured the level of insulin extracted from the pancreas and islets of *IRE1α* CKO mice by ELISA in which the amount of total proteins was used as an internal standard. In addition, we performed immunohistochemical analysis of insulin and glucagons in the tissue sections of the pancreas of *IRE1α* CKO mice. The level of insulin extracted from the pancreas and islets of *IRE1α* CKO mice was quite similar to that of control mice (Table S2). The signals of insulin and glucagons in the pancreas of *IRE1α* CKO mice were normally detected (Figure 2A). Number and size of the islets and ratio of insulin^+^ cells to glucagons^+^ cells per islet were also nearly equal between *IRE1α* CKO and control mice (Figure 2B). Thus, there is no histological difference in the islets between *IRE1α* CKO and control mice.

### IRE1α disruption causes histological abnormality of the pancreatic acinar and salivary gland serous tissues

**Figure 3:**
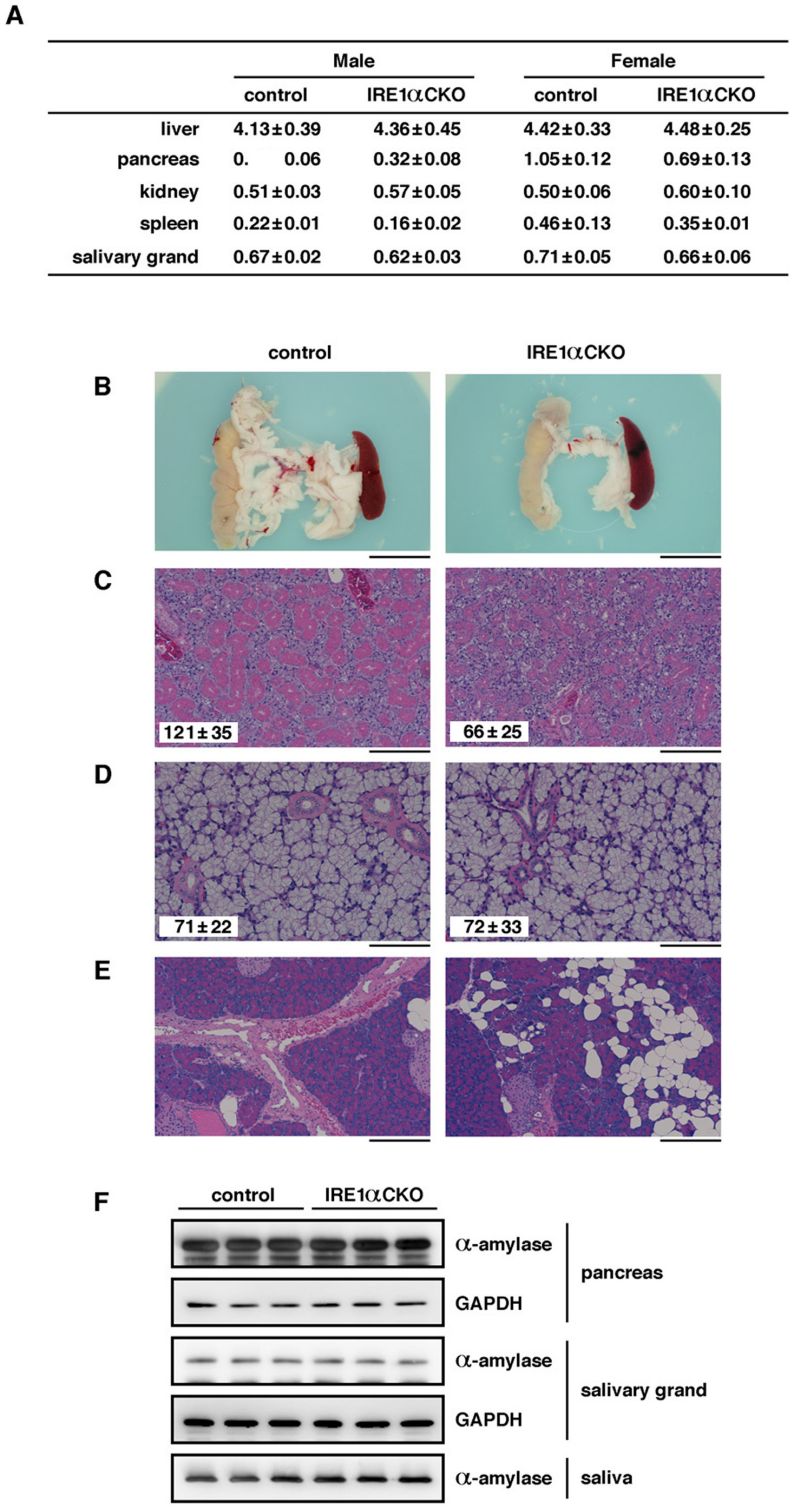
Effect of the deletion of IRE1α on the pancreatic exocrine gland and salivary glands. (**A**) Comparison of raw weight (g) of organs between the *IRE1α* CKO mice and the control mice (20 weeks old). Data are expressed as mean ± S.E.M (n = 7–10). (**B**) Pancreas collected from the mice. The pancreas come with the spleen and part of the small intestine (scale bar: 1 cm). (**C**) HE-stained sections of the serous tissue of the salivary glands (scale bar: 200 μm). The longest diameter of serous acini is shown as mean ± S.E.M (n = 50). Statistical significance of differences between control and *IRE1α* CKO mice was determined by Student's t-test (*p*<0.005). (**D**) HE-stained sections of the mucous tissue of the salivary glands (scale bar: 100 μm). The longest diameter of mucous acini is shown as mean ± S.E.M (n = 50). (**E**) HE-stained sections of the pancreatic acinar tissue (scale bar: 200 μm). (**F**) Western blot analysis of amylase in the pancreas, salivary glands and saliva. For each genotype, samples derived from three mice were used. Each saliva sample used was equal in volume. GAPDH was used as an internal standard. All the data in B-F were obtained from 20 weeks old male mice.

As described above, the embryonic lethality of *XBP1* KO mice could be rescued by the expression of a liver-specific *XBP1* transgene; however, the mice die a few days after birth. This death is thought to be caused by the dysfunction of digestion-associated exocrine glands, such as the pancreas and salivary glands [22]. Therefore, we investigated the size and histological phenotype of the pancreas and salivary glands of *IRE1α* CKO mice. We found that the size of the salivary glands of the *IRE1α* CKO mice was normal, but their pancreas was 30∼50% smaller than that of control mice (Figure 3A and 3B and Figure S1). HE-stained sections revealed that the salivary gland serous acini of the *IRE1α* CKO mice were half the size compared with those of the control mice; however, the size of the salivary gland mucous acini of the *IRE1α* CKO mice was similar to that of the control mice (Figure 3C and 3D and Figure S2 and S3). On the other hand, the pancreatic acinar tissue of the *IRE1α* CKO mice showed local spongy lesion with loss of acinar cells (Figure 3E and Figure S4), which started occurring four weeks after birth. We suspected that this pathological change was caused by apoptosis; however, TUNEL analysis showed no difference between the *IRE1α* CKO mice and the control mice (Table S3). Next, we examined the effects of *IRE1α*on the production of digestive enzymes in the pancreas and salivary glands. Amylase is one of the key digestive enzymes produced both in the pancreatic acinar and salivary gland serous cells. Western blot analysis performed using the tissue lysate and saliva revealed no difference in the amount of amylase expression and secretion between the *IRE1α* CKO mice and the control mice (Figure 3F).

### IRE1α is necessary for the differentiation of B cells into plasma cells

**Figure 4:**
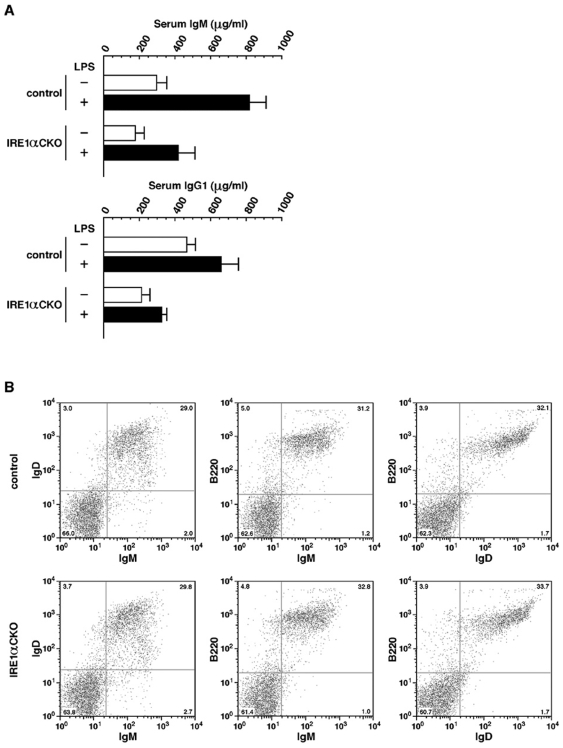
Effect of the deletion of IRE1α on B cells. (**A**) Serum immunoglobulin levels measured by ELISA. Columns indicate mean and error bars denote S.E.M (n = 5). All the data were obtained from 8 weeks old female mice. (**B**) Flow cytometry analysis of splenocytes using IgM, IgD and B220 as markers. The values at the four corners of each chart indicate the proportions of positive or negative cells for each marker. Reproducible data from three separate experiments are shown. All the data were obtained from 20 weeks old male mice.

Previous studies have demonstrated that IRE1α was activated during the differentiation of B cells into plasma cells and that XBP1 played an essential role during the same process [21], [23], [25]. To investigate the roles of IRE1α in the differentiation of B cells in mice, serum IgM and IgG1 as well as the populations of IgM^+^, IgD^+^ and B220^+^ cells in splenocytes were analyzed in the *IRE1α* CKO mice. Serum IgM and IgG1 serve as markers of plasma cell differentiation, and IgM, IgD and B220 serve as markers of mature B cells. ELISA revealed that serum levels of IgM and IgG1 were reduced by half in the *IRE1α* CKO mice compared with the control mice. Lipopolysaccharide (LPS) stimulation increases the concentration of these immunoglobulins two- to three-fold; however, as in the case of no LPS stimulation, the levels of IgM and IgG1 were approximately half in the *IRE1α* CKO mice compared with the control mice (Figure 4A). Flow cytometry analysis of splenocytes showed that the populations of positive or negative cells for IgM, IgD and B220 markers were similar between the *IRE1α* CKO mice and the control mice (Figure 4B). This suggests that IRE1α disruption causes decrease of serum level of immunoglobulin produced in the plasma cells, but does not have any effect on the differentiation into mature B cells.

### Hepatic function of IRE1α-deficient mice is normal

**Figure 5:**
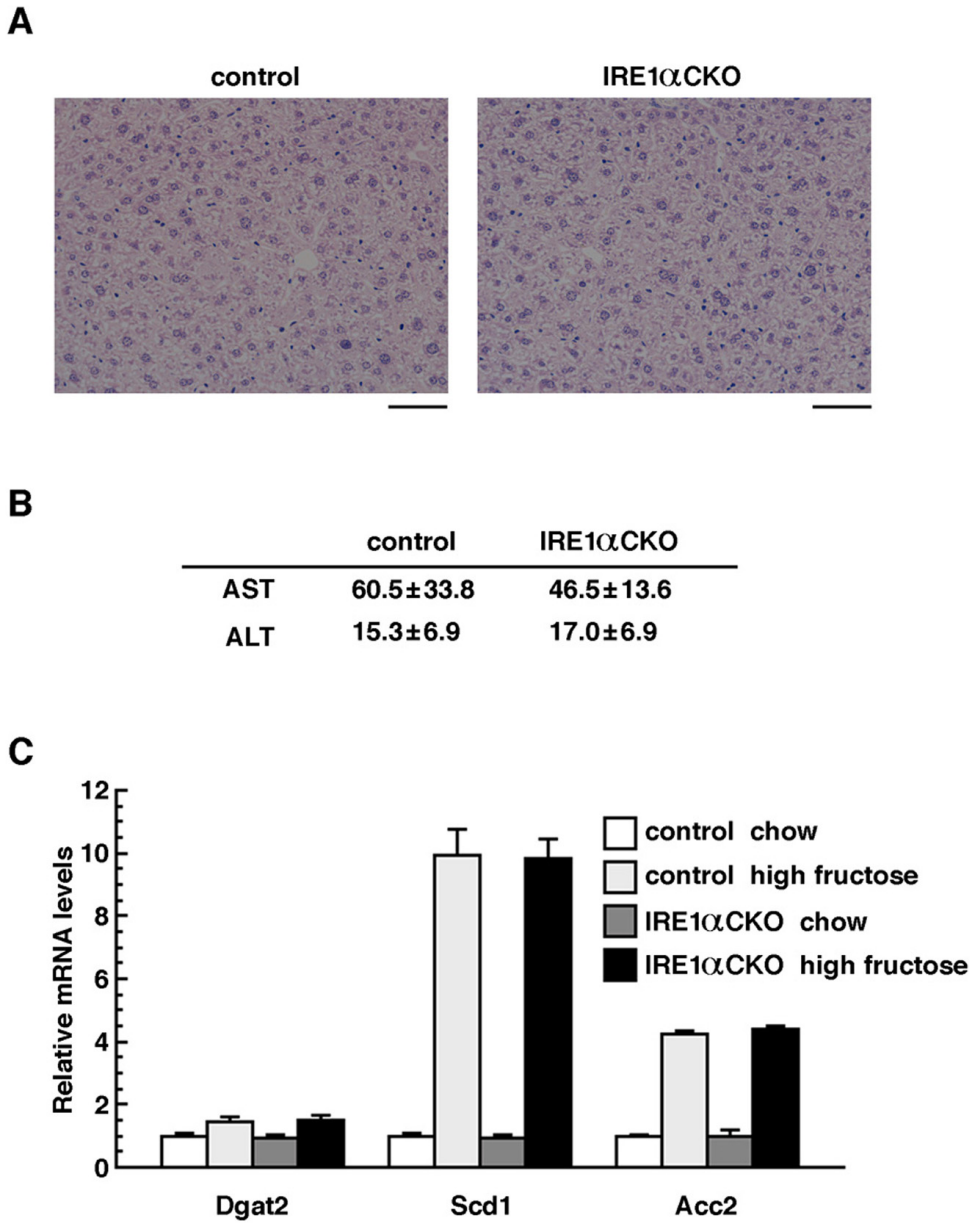
Effect of the deletion of IRE1α on the liver. (**A**) HE-stained sections of the liver tissue (scale bar: 50 μm). (**B**) Comparison of the serum AST and ALT levels between the *IRE1α* CKO mice and the control mice. Data are expressed as mean ± S.E.M (n = 5). (**C**) Quantitative PCR analysis of lipid synthesis genes in the liver of mice fed normal or high-fructose feed. Columns indicate mean and error bars denote S.E.M (n = 3). All the data were obtained from 20 weeks old male mice.

Hepatic abnormality has been reported in the embryo of both *IRE1α* KO mice and *XBP1* KO mice [19], [21]. Moreover, XBP1 has been shown as necessary for lipid metabolism in the hepatocytes of mature mice [24]. On the other hand, since histological analysis of the embryonic liver of *IRE1α* CKO mice previously failed to find abnormality [18], we performed phenotype analysis of the liver of *IRE1α* CKO mature mice by measurement of serum AST and ALT, observation of HE-stained sections, and analysis of the expression of lipid metabolism-related genes. However, no clear differences were found between the *IRE1α* CKO mice and the control mice (Figure 5).

### Functional IRE1α mRNA is significantly disrupted in *IRE1α* CKO mice

**Figure 6:**
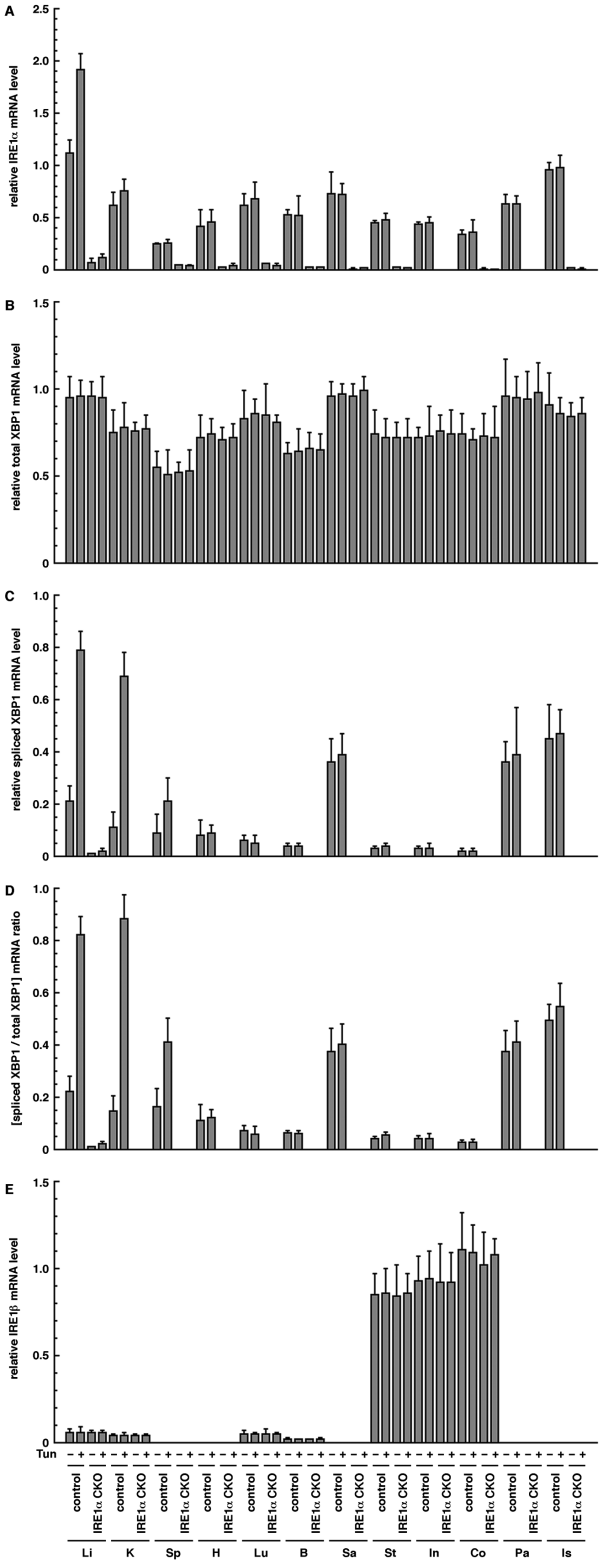
Quantitative PCR analysis of IRE1α, IRE1β, and XBP1. (**A**) IRE1α. (**B**) Total XBP1. (**C**) Spliced XBP1. (**D**) Ratio of spliced XBP1 to total XBP1. (**E**) IRE1β. Tun − or + indicates i.p. injection with saline or tunicamycin (500 ng/g body weight) 16 before tissue collection. Li; liver, K; kidney, Sp; spleen, H; heart, Lu; lung, B; brain, Sa; salivary gland, St; stomach, In; intestine, Co; colon, Pa; pancreas, Is; islet. All the data were obtained from 20 weeks old male mice.

Quantitative PCR analysis of IRE1α and XBP1 in various tissues was performed to determine the degree of functional disruption of IRE1α mRNA in the *IRE1α* CKO mice. In all the tissues tested, PCR signals of IRE1α were detected in the control mice as previously reported [18], but weakly or not detected in the *IRE1α* CKO mice (Figure 6A). Although PCR signals of total XBP1 were similarly detected in both the control and *IRE1α* CKO mice (Figure 6B), those of spliced XBP1 were slightly or obviously detected in the control mice, but little in the *IRE1α* CKO mice (Figure 6C and 6D). In the case that mice were injected with tunicamycin, an ER streesor, the spliced XBP1 level of *IRE1α* CKO mice were hardly detected in all the tested tissues, but that of control mice strongly increased in the liver and kidney and slightly increased in other tissues (Figure 6C and 6D). In addition, although IRE1β is specifically expressed in the gastrointestinal tract [26], IRE1β expression was examined in the same tissues by quantitative PCR. As expected, PCR signals of IRE1β were predominantly detected in the gastrointestinal tract in both the control and *IRE1α* CKO mice, but slightly in the other tissues (Figure 6E). These results confirm that functional IRE1α mRNA and spliced XBP1 mRNA are significantly disrupted in the *IRE1α* CKO mice and suggest that IRE1β may not complement the role of IRE1α in the *IRE1α* CKO mice. On the other hand, IRE1α mRNA and spliced XBP1 mRNA were slightly detected in some tissues of the *IRE1α* CKO mice. Thus, IRE1α is not completely disrupted in the *IRE1α* CKO mice, which may be the reason why the phenotype of *IRE1α* CKO mice is not clear-cut.

## Discussion

ER stress is associated with various diseases [27], and the *in vivo* functions of ER stress-related molecules are presently receiving attention. With regard to IRE1α the functional analysis of this molecule in adult KO mice has been lagging because the KO mice suffer embryonic lethality; however, we could show here using the conditional KO method that *IRE1α*-deficient mice exhibit mild hypoinsulinemia, hyperglycemia, and a low-weight trend, and that IRE1α disruption causes histological abnormality of the pancreatic acinar and salivary serous tissues and decrease of serum level of immunoglobulin produced in the plasma cells.

In the present study, the level of insulin protein extracted from the pancreas and islets was similar between the *IRE1α* CKO mice and the control mice. Presumably, insulin expression level is normal in *IRE1α*-deficient β cells. Therefore, the lower level of blood insulin in the *IRE1α* CKO mice may be due to an abnormality of insulin secretion. Insulin is prevented from entering its secretory pathway by the quality control of ER proteins if it does not receive proper modifications (e.g., folding and disulfide bond formation) during its biosynthesis and secretion. It is possible that IRE1α is indirectly involved with the maturation process of insulin in the ER by inducing the expression of chaperones, disulfide isomerase and other enzymes. On the other hand, based on *in vitro* analysis of the effect of IRE1α on insulin, suppression of IRE1α has been reported to cause low insulin biosynthesis at the translation level but has little impact on insulin secretion [28]. The downregulation of IRE1α has also been reported to suppress insulin mRNA degradation that occurs under a chronic high-glucose environment [29]. However, we have not yet performed experiments to confirm these findings, because it is not easy to prepare many samples and mice for detailed experiments on insulin biosynthesis; in theory, the *IRE1α* CKO mice that we used here have only a 25% chance of being born. In the future, we should aim to analyze the functions of IRE1α in association with insulin biosynthesis in the β cells of the islets by generating mice in which IRE1α is disrupted specifically in β cells. It was previously reported that loss of XBP1 had no effect on the development of the islets [22] and that *XBP1^+/−^* mice tended to have high blood glucose levels [30]. However, it remains unclear how IRE1α and XBP1 function in terms of insulin biosynthesis/secretion and the control of blood glucose level. In other words, it is uncertain whether IRE1α and XBP1 function on the same pathway or independently. To answer these questions, we need to generate mice expressing the active (spliced) form of XBP1. We believe that valuable information on the functional relationship between IRE1α and XBP1 can be obtained by analysis of insulin biosynthesis/secretion and blood glucose level in the offspring produced by crossing *IRE1α* KO mice and *XBP1* KO mice to the mice expressing the spliced XBP1.

Size reduction of acinar tissue and partial deletion of acinar cells in the pancreas and size reduction of serous acini in the salivary gland were observed in the *IRE1α* CKO mice. The pancreatic acinar tissue and salivary gland serous tissue are both representative organs for the secretion of digestive enzymes and produce a large amount of proteins such as amylase. As with insulin, amylase is prevented from entering the secretory pathway by quality control of ER proteins if it does not receive proper modifications in the ER (e.g., folding and disulfide bond formation) during its biosynthesis and secretion. IRE1α is activated in the pancreatic acinar tissue and salivary gland even under physiological conditions and excessive expression of amylase causes ER stress and IRE1α activation [31], [32]. Based on these previous findings, it is possible that IRE1α is involved in homeostasis in the ER and the development and/or survival of exocrine cells in the pancreatic acinar tissue and salivary gland by constantly inducing the expression of chaperones, disulfide isomerase and other enzymes. As described above, a developmental disorder of the pancreatic acinar tissue and salivary gland is also observed in *XBP1* CKO mice [22]. Therefore, signal transduction from IRE1α to XBP1 is important for the development and/or maintenance of the pancreatic acinar tissue and salivary gland. However, unlike in *IRE1α* CKO mice, phenotypes are more severe in *XBP1* CKO mice as exemplified by observation of apoptotic signals in the pancreatic acinar tissue, reduced production of digestive enzymes in the pancreatic acinar tissue and salivary gland, indigestion of food, and death shortly after birth [22]. This may indicate the importance of IRE1α-independent XBP1 functions for the development and/or maintenance of the pancreatic acinar tissue and salivary gland.

Like hormones (e.g., insulin and glucagons) and digestive enzymes, antibodies are also secretory proteins and are prevented from entering their secretory pathway by quality control of ER proteins if they do not receive proper modifications in the ER (e.g., folding and disulfide bond formation) during their biosynthesis and secretion. Therefore, IRE1α is believed to play an important role in plasma cells in terms of the production and quality of antibodies. In the present study, the serum levels of IgM and IgG1 in the *IRE1α* CKO mice were half of those in the control mice, and this trend was observed regardless of the presence of LPS stimulation. Previous studies also revealed IRE1α activation during the differentiation of B cells into plasma cells and that XBP1 played an essential role in the differentiation process [21], [23], [25]. Based on these findings, IRE1α may contribute to the normal production of antibodies by activating XBP1 on known UPR pathways, thereby inducing the expression of chaperones, disulfide isomerase and other enzymes in the ER.

Hepatic abnormalities in the embryos of *IRE1α* KO and *XBP1* KO mice have been reported [19], [21], while the absence of histological abnormalities in the embryonic liver of *IRE1α* CKO mice has been described [18]. Also, XBP1 has been shown to be essential for lipid metabolism in the hepatic cells of mature mice [24]. In the present study, hepatic abnormalities were not found on analysis of *IRE1α* CKO mice. This may suggest the existence of IRE1α-independent XBP1 functions. If XBP1 exerts functions depending on IRE1α, splicing of XBP1 mRNA by IRE1α would be necessary. Also, splicing of XBP1 mRNA is blocked by inactivation of IRE1α [18]. Given these observations, the functions of IRE1α-independent XBP1 may be brought about by unspliced XBP1.

Phenotype analysis of *IRE1α* CKO mice in this study provided important information regarding the physiological functions of IRE1α. Comparison of our results with the phenotypes of XBP1 KO mice previously reported indicates the possibilities that IRE1α has functions independent of XBP1 and XBP1 has functions independent of IRE1α. This study therefore provided findings that are important for revealing new roles played by IRE1α and XBP1 *in vivo*.

In this regard, however it is also possible that the discrepancies between the present data and those from Glimcher's [22], [24] and Kaufman's [21] groups may depends on difference among each experimental condition. For example, in conditional KO mice with the exogenous target gene [21], [22], the exogenous target gene may be different from the endogenous target gene in expression level and have any unexpected dominant effects, unlikely in conditional KO mice with flox/Cre system. Further, difference in mouse genetic background may raise distinct compensatory mechanism by other UPR pathways between IRE1α and XBP1 CKO mice even if these mice are commonly generated with flox/Cre system. Therefore we need to carefully analyze the phenotypes of IRE1α and XBP1 disruption under an identical experimental condition in the future.

## Supporting Information

Table S1 Body length, feed intake, and water intake.
(0.03 MB DOC)

Table S2 Insulin content in the islet and the pancreas.
(0.03 MB DOC)

Table S3 Total number of TUNEL positive cells in all acinar cells in a tissue section.
(0.03 MB DOC)

Figure S1 Fig. 3B is enlarged for the presentation of fine histological structures. Sp; spleen, In; intestine, Pa; pancreas, which is surrounded by a dashed line.
(0.62 MB TIF)

Figure S2 Fig. 3C is enlarged for the presentation of fine histological structures. Two distinctive serous acini in each panel are surrounded by a dashed line for ease of comparing the size of acini between control and IRE1α CKO mice.
(1.06 MB TIF)

Figure S3 Fig. 3D is enlarged for the presentation of fine histological structures. Two distinctive mucous acini in each panel are surrounded by a dashed line for ease of comparing the size of acini between control and IRE1α CKO mice.
(1.03 MB TIF)

Figure S4 Fig. 3E is enlarged for the presentation of fine histological structures. Ac; acinar tissue, Is; islet. The local spongy lesion (unstained area) with loss of acinar cells is impressive in pancreatic tissue of IRE1α CKO compared with that of control mice.
(0.95 MB TIF)

## References

[1] Anelli T, Sitia R (2008). Protein quality control in the early secretory pathway.. EMBO J.

[2] Ellgaard L, Helenius A (2003). Quality control in the endoplasmic reticulum.. Nat Rev Mol Cell Biol.

[3] Schröder M (2008). Endoplasmic reticulum stress responses.. Cell Mol Life Sci.

[4] Cox JS, Shamu CE, Walter P (1993). Transcriptional induction of genes encoding endoplasmic reticulum resident proteins requires a transmembrane protein kinase.. Cell.

[5] Mori K, Ma W, Gething MJ, Sambrook J (1993). A transmembrane protein with a cdc2+/CDC28-related kinase activity is required for signaling from the ER to the nucleus.. Cell.

[6] Shamu ES, Walter P (1996). Oligomerization and phosphorylation of the Ire1p kinase during intracellular signaling from the endoplasmic reticulum to the nucleus.. EMBO J.

[7] Cox JS, Walter P (1996). A novel mechanism for regulating activity of a transcription factor that controls the unfolded protein response.. Cell.

[8] Calfon M, Zeng H, Urano F, Till JH, Hubbard SR (2002). IRE1 couples endoplasmic reticulum load to secretory capacity by processing the XBP-1 mRNA.. Nature.

[9] Tirasophon W, Welihinda AA, Kaufman RJ (1998). A stress response pathway from the endoplasmic reticulum to the nucleus requires a novel bifunctional protein kinase/endoribonuclease (Ire1p) in mammalian cells.. Genes Dev.

[10] Wang XZ, Harding HP, Zhang Y, Jolicoeur EM, Kuroda M (1998). Cloning of mammalian Ire1 reveals diversity in the ER stress responses.. EMBO J.

[11] Iwawaki T, Hosoda A, Okuda T, Kamigori Y, Nomura-Furuwatari C (2001). Translational control by the ER transmembrane kinase/ribonuclease IRE1 under ER stress.. Nat Cell Biol.

[12] Yoshida H, Matsui T, Yamamoto A, Okada T, Mori K (2001). XBP1 mRNA is induced by ATF6 and spliced by IRE1 in response to ER stress to produce a highly active transcription factor.. Cell.

[13] Yoshida H, Haze K, Yanagi H, Yura T, Mori K (1998). Identification of the cis-acting endoplasmic reticulum stress response element responsible for transcriptional induction of mammalian glucose-regulated proteins.. J Biol Chem.

[14] Harding HP, Zhang Y, Ron D (1999). Protein translation and folding are coupled by an endoplasmic-reticulum-resident kinase.. Nature.

[15] Harding HP, Novoa I, Zhang Y, Zeng H, Wek R (2000). Regulated translation initiation controls stress-induced gene expression in mammalian cells.. Mol Cell.

[16] Haze K, Yoshida H, Yanagi H, Yura T, Mori K (1999). Mammalian transcription factor ATF6 is synthesized as a transmembrane protein and activated by proteolysis in response to endoplasmic reticulum stress.. Mol Biol Cell.

[17] Ye J, Rawson R B, Komuro R, Chen X, Davé UP (2000). ER stress induces cleavage of membrane-bound ATF6 by the same proteases that process SREBPs.. Mol Cell.

[18] Iwawaki T, Akai R, Yamanaka S, Kohno K (2009). Function of IRE1 alpha in the placenta is essential for placental development and embryonic viability.. Proc Natl Acad Sci U S A.

[19] Reimold AM, Etkin A, Clauss I, Perkins A, Friend DS (2000). An essential role in liver development for transcription factor XBP-1.. Genes Dev.

[20] Urano F, Wang X, Bertolotti A, Zhang Y, Chung P (2000). Coupling of stress in the ER to activation of JNK protein kinases by transmembrane protein kinase IRE1.. Science.

[21] Zhang K, Wong HN, Song B, Miller CN, Scheuner D (2005). The unfolded protein response sensor IRE1alpha is required at 2 distinct steps in B cell lymphopoiesis.. J Clin Invest.

[22] Lee AH, Chu GC, Iwakoshi NN, Glimcher LH (2005). XBP-1 is required for biogenesis of cellular secretory machinery of exocrine glands.. EMBO J.

[23] Reimold AM, Iwakoshi NN, Manis J, Vallabhajosyula P, Szomolanyi-Tsuda E (2001). Plasma cell differentiation requires the transcription factor XBP-1.. Nature.

[24] Lee AH, Scapa EF, Cohen DE, Glimcher LH (2008). Regulation of hepatic lipogenesis by the transcription factor XBP1.. Science.

[25] Iwakoshi NN, Lee AH, Vallabhajosyula P, Otipoby KL, Rajewsky K (2003). Plasma cell differentiation and the unfolded protein response intersect at the transcription factor XBP-1.. Nat Immunol.

[26] Bertolotti A, Wang X, Novoa I, Jungreis R, Schlessinger K (2001). Increased sensitivity to dextran sodium sulfate colitis in IRE1β-deficient mice.. J Clin Invest.

[27] Yoshida H (2007). ER stress and diseases.. FEBS J.

[28] Lipson KL, Fonseca SG, Ishigaki S, Nguyen LX, Foss E (2006). Regulation of insulin biosynthesis in pancreatic beta cells by an endoplasmic reticulum-resident protein kinase IRE1.. Cell Metab.

[29] Lipson KL, Ghosh R, Urano F (2008). The role of IRE1alpha in the degradation of insulin mRNA in pancreatic beta-cells.. PLoS One.

[30] Özcan U, Cao Q, Yilmaz E, Lee AH, Iwakoshi NN (2004). Endoplasmic reticulum stress links obesity, insulin action, and type 2 diabetes.. Science.

[31] Iwawaki T, Akai R, Kohno K, Miura M (2004). A transgenic mouse model for monitoring endoplasmic reticulum stress.. Nat Med.

[32] Hosoda A, Tokuda M, Akai R, Kohno K, Iwawaki T (2009). Positive contribution of ERdj5/JPDI to endoplasmic reticulum protein quality control in the salivary gland.. Biochem J.

