# Supplementary material for: IRE1α Disruption Causes Histological Abnormality of Exocrine Tissues, Increase of Blood Glucose Level, and Decrease of Serum Immunoglobulin Level
Source: PLoS One. 2010 Sep 27;5(9):e13052. doi: 10.1371/journal.pone.0013052 (PMC2946364; doi:10.1371/journal.pone.0013052)
Supplement: Table S1 — Body length, feed intake, and water intake. (0.03 MB DOC) [file pone.0013052.s001.doc]

|  |  | Male | | |  | Female | | |
| --- | --- | --- | --- | --- | --- | --- | --- | --- |
|  |  | Control |  | *IRE1α* CKO |  | Control |  | *IRE1α* CKO |
| Body length (mm) |  | 93.9±0.8 |  | 93.5±0.9 |  | 92.5±0.8 |  | 91.6±0.5 |
| Feed intake (g/day) |  | 3.6±0.2 |  | 3.8±0.2 |  | 3.4±0.2 |  | 3.3±0.1 |
| Water intake (ml/day) |  | 5.8±0.9 |  | 5.7±0.3 |  | 4.0±0.1 |  | 3.9±0.2 |

Table S1. Body length, feed intake, and water intake

Data are presented as mean ± standard deviation (n=7-10).

Measurement was performed at 17-19 weeks old.
