# Supplementary material for: IRE1α Disruption Causes Histological Abnormality of Exocrine Tissues, Increase of Blood Glucose Level, and Decrease of Serum Immunoglobulin Level
Source: PLoS One. 2010 Sep 27;5(9):e13052. doi: 10.1371/journal.pone.0013052 (PMC2946364; doi:10.1371/journal.pone.0013052)
Supplement: Table S2 — Insulin content in the islet and the pancreas. (0.03 MB DOC) [file pone.0013052.s002.doc]

|  |  | Male | | |  | Female | | |
| --- | --- | --- | --- | --- | --- | --- | --- | --- |
|  |  | Control |  | *IRE1α* CKO |  | Control |  | *IRE1α* CKO |
| Insulin/islet (ng/islet) |  | 70±16 |  | 76±12 |  | 62±20 |  | 59±15 |
| Insulin/pancreas (μg/g) |  | 823±36 |  | 884±41 |  | 769±34 |  | 732±29 |

Table S2. Insulin content in the islet and the pancreas

Data are presented as mean ± standard deviation (n=7-10).

Measurement was performed at 20 weeks old.
