# Supplementary material for: IRE1α Disruption Causes Histological Abnormality of Exocrine Tissues, Increase of Blood Glucose Level, and Decrease of Serum Immunoglobulin Level
Source: PLoS One. 2010 Sep 27;5(9):e13052. doi: 10.1371/journal.pone.0013052 (PMC2946364; doi:10.1371/journal.pone.0013052)
Supplement: Table S3 — Total number of TUNEL positive cells in all acinar cells in a tissue section. (0.03 MB DOC) [file pone.0013052.s003.doc]

| Male | | |  | Female | | |
| --- | --- | --- | --- | --- | --- | --- |
| Control |  | *IRE1α* CKO |  | Control |  | *IRE1α* CKO |
| 15 |  | 17 |  | 18 |  | 12 |

Table S3. Total number of TUNEL positive cells in all acinar cells in a tissue section

Measurement was performed at 20 weeks old.
